# Effects of Inhibiting Dipeptidyl Peptidase-4 (DPP4) in Cows with Subclinical Ketosis

**DOI:** 10.1371/journal.pone.0136078

**Published:** 2015-08-20

**Authors:** Kirsten Schulz, Jana Frahm, Susanne Kersten, Ulrich Meyer, Jürgen Rehage, Marion Piechotta, Maria Meyerholz, Gerhard Breves, Dania Reiche, Helga Sauerwein, Sven Dänicke

**Affiliations:** 1 Institute of Animal Nutrition, Federal Research Institute for Animal Health, Friedrich-Loeffler-Institute, Braunschweig, Germany; 2 Clinic for Cattle, University of Veterinary Medicine Hannover, Hannover, Germany; 3 Department of Physiology, University of Veterinary Medicine Hannover, Hannover, Germany; 4 Boehringer Ingelheim Vetmedica, Ingelheim am Rhein, Germany; 5 Physiology and Hygiene Unit, Institute of Animal Science, University of Bonn, Bonn, Germany; Max-Delbrück Center for Molecular Medicine (MDC), GERMANY

## Abstract

The inhibition of dipeptidyl peptidase-4 (DPP4) via specific inhibitors is known to result in improved glucose tolerance and insulin sensitivity and decreased accumulation of hepatic fat in type II diabetic human patients. The metabolic situation of dairy cows can easily be compared to the status of human diabetes and non-alcoholic fatty liver. For both, insulin sensitivity is reduced, while hepatic fat accumulation increases, characterized by high levels of non-esterified fatty acids (NEFA) and ketone bodies.Therefore, in the present study, a DPP4 inhibitor was employed (BI 14332) for the first time in cows. In a first investigation BI 14332 treatment (intravenous injection at dosages of up to 3 mg/kg body weight) was well tolerated in healthy lactating pluriparous cows (n = 6) with a significant inhibition of DPP4 in plasma and liver. Further testing included primi- and pluriparous lactating cows suffering from subclinical ketosis (β-hydroxybutyrate concentrations in serum > 1.2 mM; n = 12). The intension was to offer effects of DPP4 inhibition during comprehensive lipomobilisation and hepatosteatosis. The cows of subclinical ketosis were evenly allocated to either the treatment group (daily injections, 0.3 mg BI 14332/kg body weight, 7 days) or the control group. Under condition of subclinical ketosis, the impact of DPP4 inhibition via BI 14332 was less, as in particular β-hydroxybutyrate and the hepatic lipid content remained unaffected, but NEFA and triglyceride concentrations were decreased after treatment. Owing to lower NEFA, the revised quantitative insulin sensitivity check index (surrogate marker for insulin sensitivity) increased. Therefore, a positive influence on energy metabolism might be quite possible. Minor impacts on immune-modulating variables were limited to the lymphocyte CD4^+^/CD8^+^ ratio for which a trend to decreased values in treated versus control animals was noted. In sum, the DPP4 inhibition in cows did not affect glycaemic control like it is shown in humans, but was able to impact hyperlipemia, as NEFA and TG decreased.

## Introduction

Dipeptidyl peptidase-4 (DPP4) plays a major role in glucose metabolism and is responsible for the degradation of incretin hormones, such as glucagon-like peptide-1 (GLP-1). Human GLP-1 is released from the small intestine in response to oral glucose [1]. It stimulates insulin secretion via activating specific receptors on the islet β-cells, suppresses glucagon secretion, inhibits gastric emptying and reduces appetite [2]. Furthermore, chronic elevated concentrations of GLP-1 were shown to result in reduced hepatic fat accumulation and significantly lower TG concentrations in rat and mouse model [3]. However, after enzymatic degeneration via DPP4, which occurs within minutes following ingestion, only 10–20% of active GLP-1 remains in blood. Today, DPP4 inhibitors are employed in human medicine to prolong the beneficial incretin effects, in particular to improve insulin sensitivity, with the aim to treat type II diabetes [4].

In high-yielding dairy cows, the metabolic status around calving and the onset of lactation exhibits huge parallels to patients suffering from type II diabetes and non-alcoholic fatty liver diseases, as comprehensive physiological challenges are necessary to coordinate the metabolic alterations. In the transition from late pregnancy and early lactation, decreases in insulin concentration and peripheral insulin responsiveness suppress glucose consumption by peripheral, insulin-dependent tissues (skeletal muscle, adipose tissue) and thus enhance the availability of glucose for the insulin-independent mammary gland [5]. The adaption to the negative energy balance (NEB) is often related to metabolic dysfunctions, such as excessive lipid accumulation in the liver and ketosis [6–8], characterized by increased concentrations of non-esterified fatty acid (NEFA) and β-hydroxybutyrate (BHB). Due to the infiltration of fat, lesions in hepatic tissues appear and cause increased blood levels of specific enzymes, such as γ- glutamyl transferase (γ-GT), aspartate transaminase (AST) or glutamate dehydrogenase (GLDH) [9]. Furthermore, a fatty liver contributes the development of hepatic insulin resistance and influences body´s immune system negatively. In particular, the impacts of tumor necrosis factor-α and acute phase protein reactions are well studied in cows with fatty liver and its role in immune response [10,11].

Little is known about the metabolism of incretins and its interaction with DPP4 in ruminants. In contrast to monogastric species, the cow does not rely on glucose absorption in the small intestine but uses short chain fatty acids from ruminal fermentation for her energy supply with propionate as main substrate for gluconeogenesis. However, increasing dietary energy supply has been shown to enhance the secretion of GLP-1 in steers [12] and abomasal infusion of lipid and casein, but not glucose, increased the GLP-1 concentration in cows [13,14]. The fat-induced elevation in circulating GLP-1 is believed to play a role in the short-term control of feed intake in cattle [14,15], but the wide range of tissues expressing the GLP-1 receptor (gut segments, pancreas, spleen and kidney) suggest that GLP-1 may have multiple physiological functions beyond the control of feed intake [16]. The DPP4 expression and the circulating GLP-1 concentrations in blood depend on stage of lactation. While GLP-1 concentrations increase with onset of lactation, the expression of DPP4 decreases [13,17].

Taking the background information into consideration, DPP4 is a key enzyme in intermediary metabolism by regulating important glycemic pathways. Therefore, it was possible that DPP4 inhibitors could counteract typical ketotic processes in the dairy cow. Within the present research a DPP4 inhibitor (BI 14332) was employed to regulate typically increased parameters of bovine ketosis to the physiological range, respectively to compensate a distinct NEB. Therefore, we first established appropriate dosage of BI 14332 to effectively decrease DPP4 activity in plasma and liver from healthy lactating dairy cows. The second aim was to verify the efficacy of the derived dose and dosing regimen in cows with subclinical ketosis based on evaluation of various endpoints, such as clinical-chemical parameters and immune traits as well as liver lipid concentration.

## Materials and Methods

### Ethic statement

The experiments were approved by the competent authority, the lower Saxony state office for consumer protection and food safety (LAVES; Trial 1: file no. 33.9-42502-05-11A172, Trial 2: file no. 33.14-42502-04-11/0444; Oldenburg, Germany). The regulations of the German Animal Welfare Act (TierSchG) in its respective edition were met.

### Experimental design

The investigations about the pharmacokinetics and pharmacodynamics (PK/PD) of BI 14332 were performed at the Clinic for Cattle, University of Veterinary Medicine in Hannover, Germany (Trial 1). The experiment aimed in evaluating the effectiveness of DPP4 inhibition in dairy cows with subclinical ketosis was carried out at the experimental station of the Institute of Animal Nutrition, Friedrich-Loeffler-Institute (FLI) in Braunschweig, Germany (Trial 2).

#### Trial 1

Six lactating and clinically healthy German Holstein cows (pluriparous) were treated with three different doses, i.e. 0.3, 1.0 and 3.0 mg/kg body weight (BW; injection volume: 0.01–0.1 mL/kg) of BI 14332 (*n* = 2/dosage; i.v.).

To evaluate the concentration of BI 14332 and the DPP4 activity in plasma, samples were collected 24 h before the injection, 0, 0.25, 0.5, 1, 2, 4, 6, 12, 24, and 48 h after the injection. In addition, liver samples were collected according to Starke et al. [18] 24 h before injection and 4, 24 and 48 h thereafter to evaluate the hepatic DPP4 activity.

For an in vitro activity assay, potassium EDTA plasma samples of three healthy Holstein Frisian cows were incubated with 0, 1, 3, 10, 30 and 100 nM BI 14332.

#### Trial 2

Using an animal model in which subclinical ketosis is induced [19]; the impact of DPP4 inhibition via BI 14332 on metabolic variables and on immune function was investigated. β-hydroxybutyrate concentrations between 1.2–2.5 mM in blood serum were defined as a subclinical ketotic status [20].

The chemical compositions of concentrate and total mixed ration are shown in Table 1. For more details regarding feeding management refer to Schulz et al. [19]. In brief, 20 pregnant and healthy German Holstein cows with a mean body condition score (BCS) of 3.16 ± 0.06 [21] were assigned to the experimental group. For the last six weeks of parturition, cows were fed with a high energetic ration (7.7 MJ NEL/kg dry matter). The allocation of cows with a BCS of at least 3.0 and a high energetic energy supply ante partum resulted in a higher condition at the time of calving (called “higher condition” cows; HC). The aim was to enhance lipomobilisation post partum. Immediately after calving, the energy supply by concentrate feeding was reduced first and raised stepwise (from 30% to 50% for the first three weeks of lactation).

**Table 1:** Ingredients and chemical compositions of concentrate and total mixed ration.

	Ante partum diet^a^		Post partum diet^b^	
	Concentrate	TMR	Concentrate	TMR
**Ingredients, %**				
Wheat	41.0		41.0	
Dried sugar beet pulp	30.5		30.3	
Rapeseed meal	20.0		20.0	
Soybean meal	6.5		6.5	
Vitamin/mineral premix	2.0^c^		2.0^d^	
Calcium carbonate	-		0.2	
Dry matter (DM), g/kg	877	489	875	393
**Nutrients [g/kg DM]**				
Crude ash	58	55	62	56
Crude protein	197	140	202	122
Ether extract	27	33	28	32
Crude fibre	101	163	72	194
Acid detergent fibre (ADF)	136	199	96	222
Neutral detergent fibre (NDF)	279	394	222	431
**Energy** ^e^ **, MJ NEL/kg DM**	**8.6**	**7.7**	**8.7**	**7.0**

^a^Total mixed ration (TMR) on dry matter (DM) basis (40% roughage (75% corn silage, 25% grass silage) + 60% concentrate.

^b^TMR on DM basis (70% roughage (75% corn silage, 25% grass silage) + 30% concentrate.

^c^Per kg of mineral feed: 10g Ca, 60g P, 120g Na, 60g Mg, 800,000 IU vitamin A, 100,000 IU vitamin D_3_, 2500mg vitamin E, 4000mg Mn, 6000 mg Zn, 1250mg Cu, 100mg I, 35mg Co, 50mg Se

^d^Per kg of mineral feed: 170g Ca, 50g P, 120g Na, 45g Mg, 800,000 IU vitamin A, 100,000 IU vitamin D_3_, 4000mg vitamin E, 4000mg Mn, 6000mg Zn, 1300mg Cu, 120mg I, 35mg Co, 40mg Se

^e^Calculation based on nutrient digestibilities masured with wethers (GfE, 1991) and values from feed tables (DLG, 1997)

Two cows from HC group were excluded from the experiment because of health problems, which were not due to the experimental design. Out of the 18 HC cows, 12 cows developed subclinical ketosis (serum BHB concentration ≥ 1.2 and < 2.5 mM). Six HC cows were treated with BI 14332 (HC-BI) over a period of 7 days (daily i.v. injections, 0.3 mg/kg BW/day) and the remaining six subclinical cows formed the control group (HC-Con) without treatment. Subclinical ketosis was diagnosed on day +3 (1 HC-Con cow), day +7 (5 HC-BI cows and 3 HC-Con cows) or on day +10 (1 HC-BI cow and 2 HC-Con cows), relative to calving. The remaining six HC cows were either affected with clinical ketosis (n = 3, BHB in serum > 2.5 mM) or stayed apparently healthy (BHB < 1.2 mM, n = 3). Blood samples were collected at day “0”, i.e. 48 ± 7.2 days before expected calving, and then on day -14, -7, -3, +1, +3, +7, +10, +14, +17, +21, +24, +28, +35, +42 and +56 (relative to calving) from the *V*. *jugularis*; liver biopsies were taken on day -14, +7, +21, +35 and +56.

### Sample Preparation and Analysis

#### Trial 1

**Fig 1:**
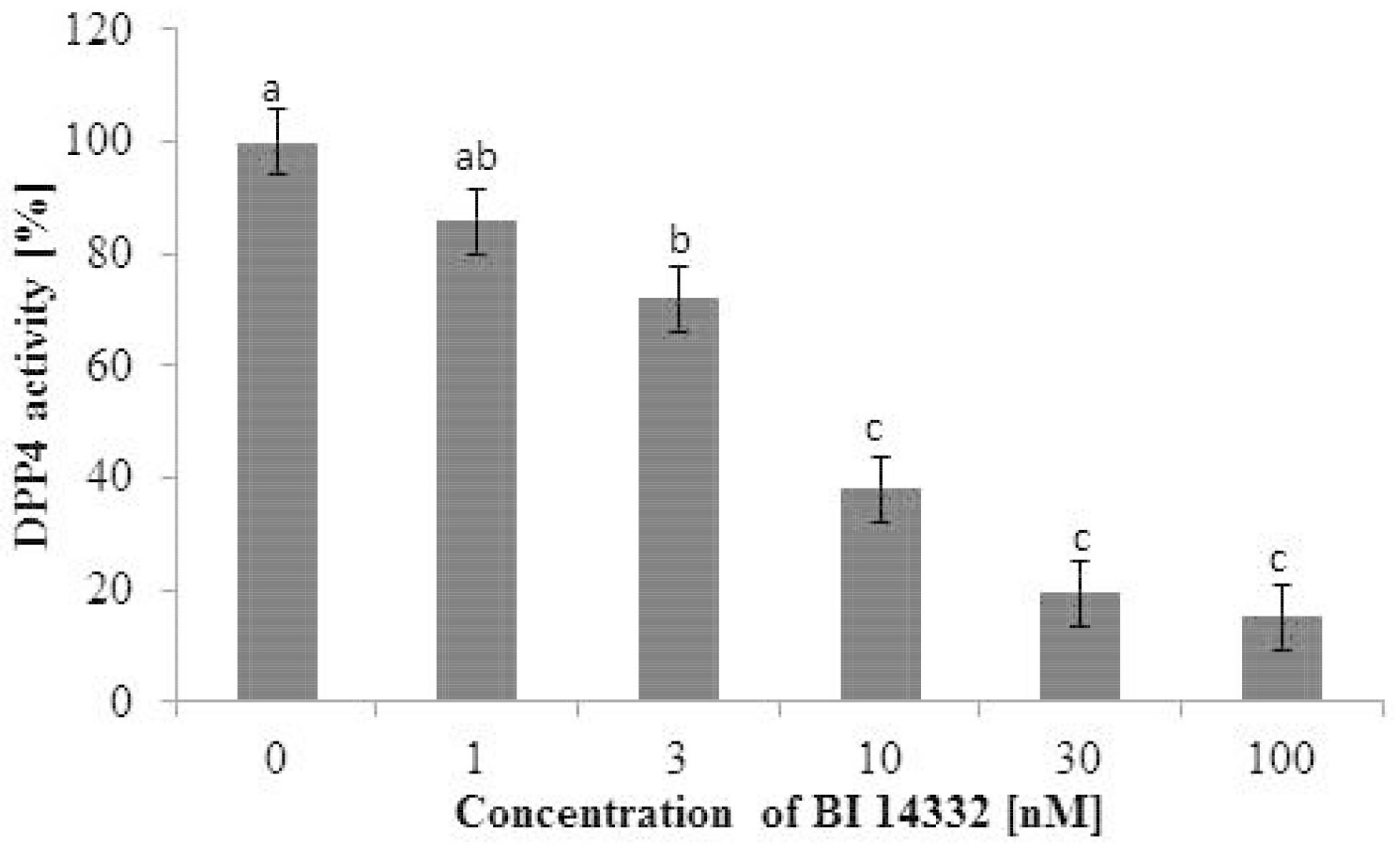
Dipeptidyl peptidase-4 (DPP4) activity assessed in vitro. Potassium EDTA plasma samples of three healthy dairy cows were incubated with different concentrations of BI 14332 (0, 1, 3, 10, 30 and 100 nM; 469 g/mol). The concentration of BI 14332 was significant (*P* = 0.006). a, b, c: Different letters indicate significant differences between dosages (*P* < 0.05, Tukey test).

The DPP4 activity in plasma and liver was assessed by a semi-quantitative assay with fluorescence detection at the Institute for Clinical Research and Development (Mainz, Germany). The method was validated for the detection of DPP4 activity in human plasma samples [22]. The dose dependently inhibition of DPP4 by increasing BI 14332 in nanomolar concentrations (Fig 1) showed that DPP4 activity in bovine samples is detectable by the method as well. The fluorescence measured as relative fluorescence units (RFU) is equivalent to the DPP4 activity in the sample. The baseline/pre-dose activity was set to 100% and all other activities measured in blood samples of the individual cows were calculated as the respective percentage of the baseline DPP4 activity.

Homogenized liver samples (20 mg) were mixed with 450 μL DPP4 tissue buffer (25 mM HEPES, 140 mM NaCl, 80 mM MgCl_2_ and 11.25 mM Triton x-100). The DPP4 tissue buffer containing liver material was collected in a vial, centrifuged (1780 g, 10 min, 4°C) and the resulting supernatant was stored at -80°C until analysis for DPP4 activity.

The concentration of BI 14332 in blood plasma was analyzed by the Pharmacelsus GmbH (Saarbrücken, Germany) via LC-MS/MS (Surveyor MS Plus HPLC system, Thermo Fisher Scientific), connected to a TSQ Quantum Discovery Max (Thermo Fisher Scientific) triple quad mass spectrometer. Data handling was done using the standard software Xcalibur 2.0.7.

#### Trial 2

Clinical chemistry [BHB, NEFA, triglycerides (TG), glucose, γ-glutamyl transferase (γ-GT), aspartate transaminase (AST), glutamate dehydrogenase (GLDH)] was assessed in serum using photometric methods (Eurolyser, Type VET CCA, Eurolyser Diagnostica GmbH). A radioimmunoassay was used to quantify the serum insulin concentrations (IM3210, Insulin IRMA KIT, Immunotech, Beckman Coulter). This immunoradiometric test was a “sandwich” type assay. The antibodies used (mouse monoclonal) were directed against two different insulin epitopes. The assay was performed according to the manufacturer’s instructions. The intra-assay CV was 7.6%, and the inter-assay CV was 10.7%. The lowest detection limit was 3.95 μU/ml. Haptoglobin (Hp) was measured by ELISA as described elsewhere [23] and the total lipid content in liver samples (~100 mg) was assessed using a gravimetrical method [18].

The daily dry matter intake (DMI) was recorded for the whole experimental time (computerized feeding station: Type RIC, Insentec). Milking took place twice a day at 05.30 and 15.30. Milk yield was recorded using automatic milk counters (Lemmer Fullwood GmbH).

Hematological analyses were performed in EDTA whole blood using an automatic analyzer (Celltac α MEK-6450, Nihon Kohden, Qinlab Diagnostik).

Functional tests (*ex vivo*) of peripheral blood mononuclear cells (PBMC) were performed in samples from days -14, +7, +10, +14, +21 and +56 (relative to calving) using the Alamar Blue assay (AB). Concanavalin A (ConA, 2.5 μg/mL final, Sigma-Aldrich) was used as mitogen to stimulate T-lymphocytes. Further details were described previously [24].

For the calculation of CD4^+^/CD8^+^ T-cell population and its CD4^+^/CD8^+^ ratio from data generated by flow cytometry, days relative to calving where pooled in accordance to treatment [day “0” and day -14 (“ante-partum”), two days of treatment (“treatment”), after treatment, i.e. day +17 until day +28 post-partum (“2 weeks post treatment”) and day +35, +42 and +56 post-partum (“end of trial”)]. A detailed description of the measurements is provided by Stelter et al. [25]. Samples were double stained with monoclonal antibodies for CD4^+^ (mouse anti bovine CD4:FITC) and CD8^+^ (mouse anti bovine CD8:RPE) or the corresponding isotype controls (mouse IgG2a negative control: RPE and mouse IgG2b negative control: FITC; all AbD Serotec).

### Statistics and Calculations

#### Trial 1

The pharmacokinetic parameters were performed using non-linear regression via STATISTICA 10 [26]. The time course of plasma concentration of BI 14332, *C*_*p*_, was expressed by a sum of two exponential functions:

$${\text{C}}_{p}=\overset{n}{\underset{i=1}{\sum }}a_{i}{\;\text{e}}^{-b_{i}t}$$

where *a*_*i*_ and *b*_*i*_ are hybrid coefficients and exponential terms, *t* is time, and *n* is the number of exponential terms. From the data obtained, area under the concentration-time curves from 0 to 24 h (AUC), terminal half-life (t_1/2_), total body clearance from 0 to 24 h (Cl_24h_), the Volume of distribution (V_d_), and the average steady state concentration (C_ss_) were calculated.

Area under the curve of DPP4 activity in plasma and liver from 0 to 24 h was calculated using the linear trapezoidal rule:

$$AUC=\overset{N}{\underset{n=1}{\sum }}\frac{C_{n}+C_{n+1}}{2}\;\left(t_{n+1}-t_{n}\right)$$

#### Trial 2

Insulin sensitivity was estimated by the Revised Quantitative Insulin Sensitivity Check Index (RQUICKI) [27]:

$$\text{RQUICKI}=\frac{1}{\text{log}\;\text{Insulin}[\mu \text{U}/\text{mL}]+\text{log}\;\text{Glucose}\;[\text{mg}/\text{dL}]+\text{log}\text{NEFA}\;[\text{mmol/L}]}$$

The results of the ex vivo examinations of PBMC were expressed as stimulation index (SI), defined as ratio between the fluorescence in the AB assay of ConA stimulated and nonstimulated PBMC:

$$\text{SI}=\frac{\text{Fluorescence of ConA stimulated PBMC}}{\text{Fluorescence of nonstimulated PBMC}}$$

For statistical analyses the SAS software package [28] was used. Evaluation of goodness of fit was carried out using the corrected Akaike information criterion. All parameters evaluated were compared as dependent variable by the MIXED procedure with a compound symmetry covariance structure. Treatment (HC-Con vs. HC-BI) was considered as fixed factor and sampling dates (time) as a repeated effect, and their respective interaction were included into the model. All results are presented as least square means (LSmeans) and standard errors (SE). Effects were declared to be significant when *P*-values were ≤ 0.05 after Tukey test for post-hoc analysis, whereas a trend was noted when 0.05 < *P* < 0.10.

Data evaluation of hematology, proliferative capability (SI) and clinical chemistry based on pooled sampling days (“week of treatment/observation”, “1^st^ week after treatment/observation” and “2^nd^ week after treatment/observation”). The day with first occurrence of serum BHB concentration ≥ 1.2 mM (day of classification) was set as covariate. Data evaluation of milk yield and DMI based on weekly mean values. The first week of lactation was set as covariate. For the proliferative capability, the SI of day +7 post partum was set as covariate. The remaining variables (liver lipid content, parameters of glycemic control, phenotyping T-lymphocytes) were analyzed in accordance to the evaluated sampling days, as described above.

## Results

### Trial 1

#### Investigations in vitro

The DPP4 activity in EDTA plasma samples decreased significantly starting at a concentration of 3 nM (≙ 1.407 ng/mL) BI 14332 as shown in Fig 1. At 100 nM (≙ 46.9 ng/mL) the remaining DPP4 activity was 15.1%.

#### Pharmacokinetics and pharmacodynamics

**Fig 2:**
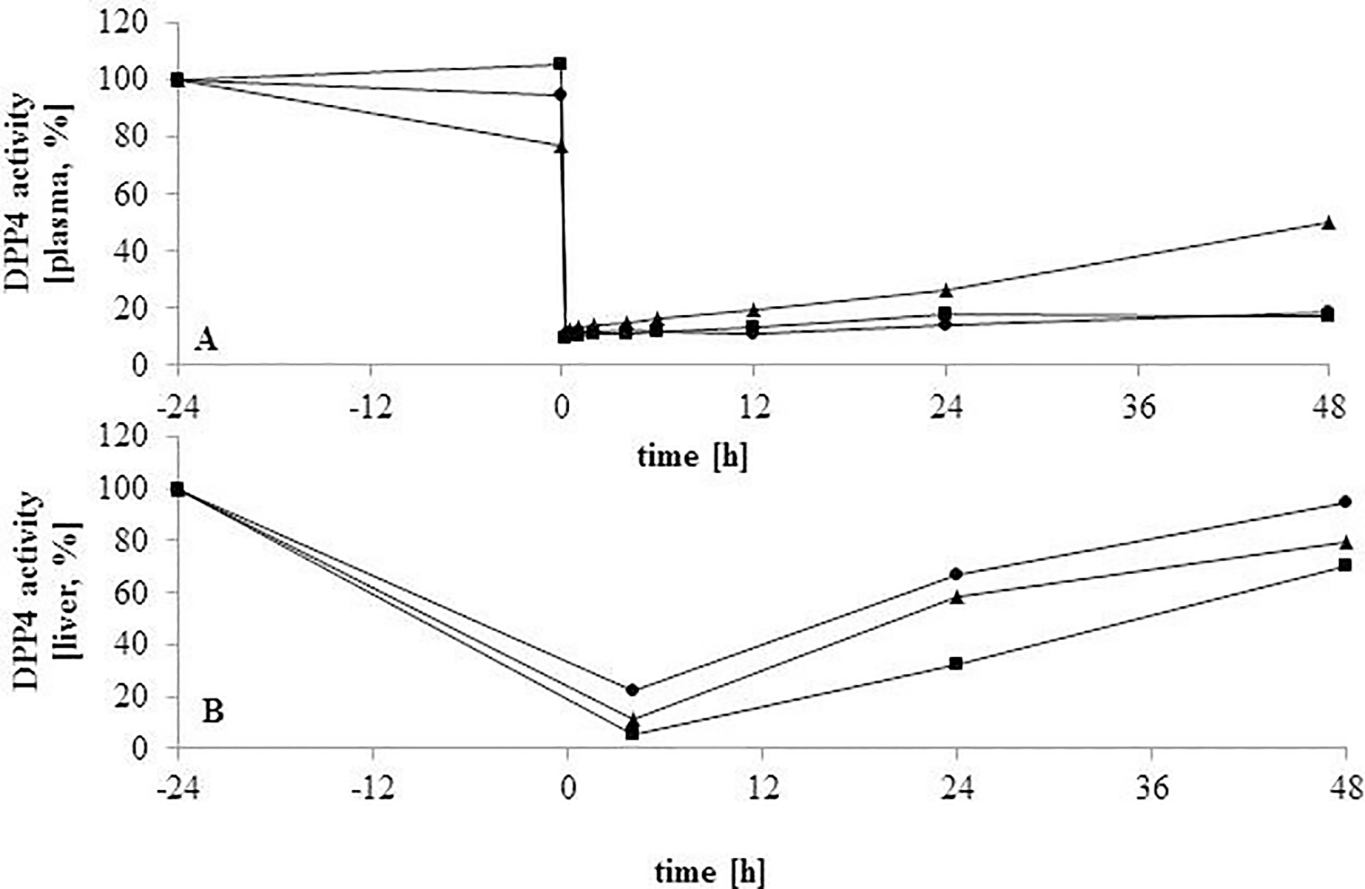
Inhibition of plasma and liver dipeptidyl peptidase-4 (DPP4) activities after injection of BI 14332. BI 14332 was administered in a single dose of 3 [square], 1 [triangle] and 0.3 [circle] mg/kg body weight in dairy cows (n = 2/group). Plasma samples (*V*. *jugularis*) were taken 24 h before and immediately before (time zero “0”) injection, as well as 0.25, 0.5, 1, 2, 4, 6, 12, 24 and 48 h post injection (upper shape). Liver was biopsied 24 h before injection, as well as 4, 24 and 48 h thereafter (lower shape).

The single administration of BI 14332 at 0.3, 1.0 or 3.0 mg/kg BW was well tolerated and a clear BI 14332 plasma concentration-dependent inhibition of the DPP4 activity both in plasma and liver was noted (Fig 2). The PK/PD variables of BI 14332 and DPP4 activity in plasma and liver are represented in Table 2. The AUC regarding BI 14332 in plasma were dose-dependently increased. The t_1/2_ of BI 14332 was highest for the 0.3 mg/kg BW dosage group, with a 10.5 to 23 h range. The V_d_ and Cl_24h_ were greatest when 3 mg/kg BW was applied. The C_ss_ decreased dose-dependently, starting with the highest dosage of BI 14332. Plasma DPP4 activity (Fig 2A) was significantly inhibited by BI 14332 at all dosages with a remaining maximum activity of 14%, which was in line with an inhibitory power of 86% (1 mg/kg BW; 15 min after injection). The single dose of 1 mg/kg also showed the lowest inhibition at 24 h after injection (~ 70%), while inhibition by the other two dose groups were greater and quite similar (81–87%). Forty-eight hours after injection, the cows treated with 0.3 and 3 mg/kg still had an inhibition of DPP4 activity of about 82% versus 74% for the 1 mg/kg dosage group (*P* < 0.05). In liver, the AUC of DPP4 activity was decreased with increasing dosage (Fig 2B).

**Table 2:** Pharmacokinetic parameters of BI 14332 und Dipeptidy peptidase-4 (DPP4) in plasma and liver of six healthy German Holstein cows treated with different dosages of BI 14332 [3, 1 and 0.3 mg/kg body weight (BW); n = 2/dosage group]^a^.

BI 14332	Dosage								
	3 mg/kg BW^b^			1 mg/kg BW^b^			0.3 mg/kg BW^b^		
	Cow 1	Cow 2	Mean	Cow 3	Cow 4	Mean	Cow 5	Cow 6	Mean
**Plasma**									
AUC_24h_ [ng•h/mL]	2076	2784	2430	1769	773	1271	809	783	796
t_1/2_ [min]	198	289	243	116	173	144	630	1386	1008
V_d_ [L/kg BW]	17.07	30.13	23.60	5.21	12.73	8.97	13.37	20.56	16.97
Cl_24h_ [mL/kg/min]	24.09	17.96	21.02	9.42	21.55	15.49	6.18	6.39	6.28
C_ss_ [ng/mL]	86.49	115.99	101.24	71.94	30.70	51.32	33.69	32.63	33.16
**DPP4**									
**Plasma**									
AUC_24h_ [RFU/h]	20491	16560	18525	21034	20135	20584	15729	17914	16822
Δ_15min_ [%]	90	91	91	86	86	86	88	90	89
Δ_24h_ [%]	81	85	83	66	73	70	84	87	85
**Liver** ^**c**^									
AUC_24h_ [μg•h/mL]	41	74	43	90	45	68	63	112	101
Δ_4h_ [%]	96	94	94	89	89	89	77	79	78
Δ_24h_ [%]	65	71	68	40	44	42	35	31	33

^a^Pharmacokinetic parameters of BI 14332 were evaluated via bi-exponential function [26]; AUC for DPP4 activity in plasma and liver was calculated using the trapezoidal rule.

^b^BI 14332 was administrated intravenously (i.v.); plasma samples were taken 24 h before injection, 0, 0.25, 0.5, 1, 2, 4, 6, 12, 24 and 48 h after injection; liver samples were taken 24 h before injection and 4, 24 and 48 h thereafter.

^c^DPP4 activity in liver was normalized to the total protein content of the samples.

AUC, area under the curve from 0 to 24 h; t_1/2_, terminal half-life; V_d_, Volume of distribution; Cl_24h_, Clearance from 0 to 24 h; C_ss_, average steady state concentration; Δ_15min_/Δ_4h_/Δ_24h_: Inhibitory power of BI 14332 regarding DPP4 activity, calculated as difference before BI 14332 application and the first sample post injection (i.e. 15 min post injection in plasma and 4 h post injection in liver) and 24 h after injection, respectively.

**Fig 3:**
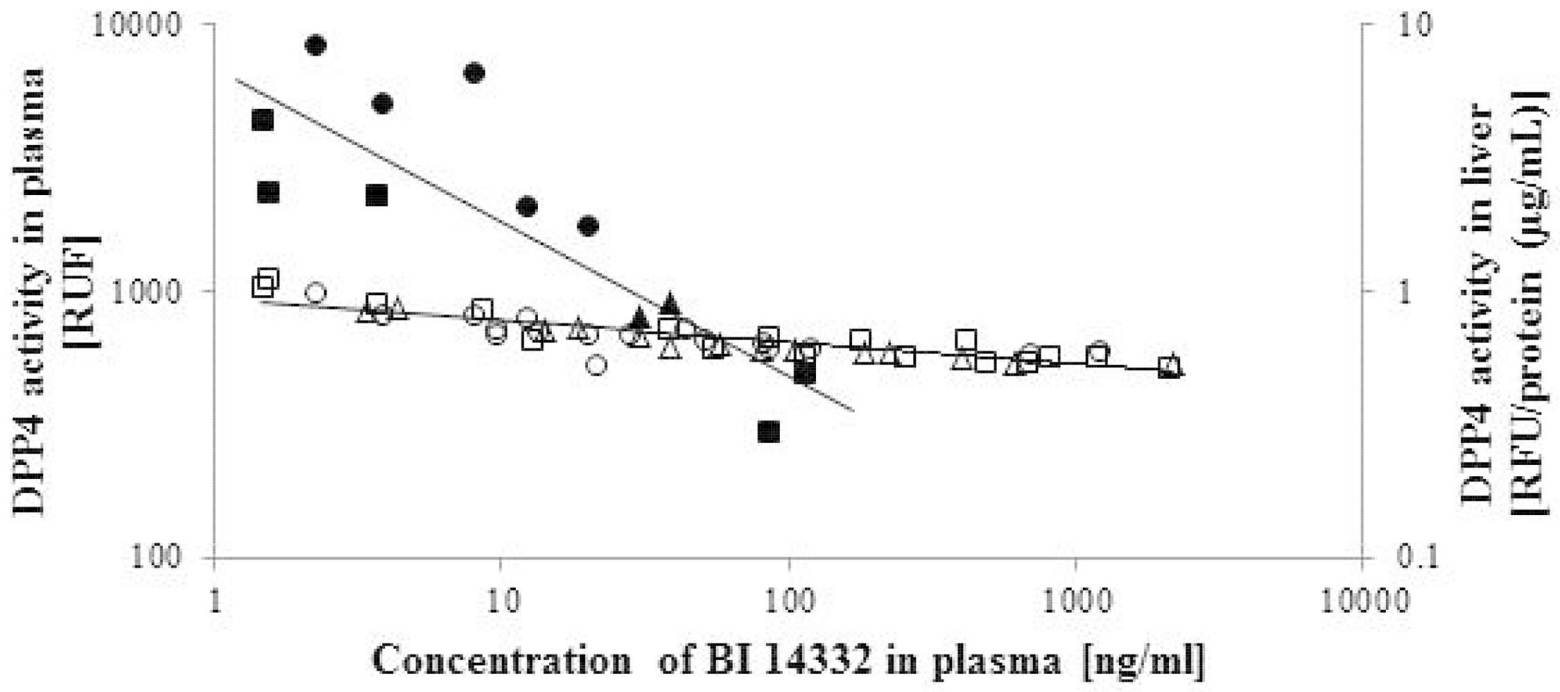
Inhibition of dipeptidyl peptidase-4 (DPP4) activity in plasma and liver after injection of BI 14332. BI 14332 was administered in a single dose of 3, 1 and 0.3 mg/kg body weight (BW) in dairy cows (n = 2/group). Plasma samples were taken 0.25, 0.5, 1, 2, 4, 6, 12, 24 and 48 h after the injection (*V*. *jugularis*; 3 mg/kg BW [□]; 1 mg/kg BW [Δ]; 0.3 mg/kg BW [○]). Liver samples were obtained by biopsy 4, 24 and 48 h after the injection (3 mg/kg BW [square]; 1 mg/kg BW [triangle]; 0.3 mg/kg BW [circle]). BI 14332 (x-axis) was shown to have a strong negative impact on DPP4 activity (y-axes), well approximated by a power function (represented as quasi linear model via log-log transformation) in liver: y = 7.72x^-0.589^ (r^2^ = 0.72) and plasma: y = 935.31x^-0.081^ (r^2^ = 0.76).

The relationship between the BI 14332 concentration in plasma and the corresponding DPP4 activity in plasma and liver is depicted in Fig 3, well approximated by a power function. With increasing plasma concentrations of BI 14332 (x-axis), a negative slope for DPP4 activity (y-axes) was observed in liver: y = 7.72x^-0.589^ (r^2^ = 0.72) and in plasma: y = 935.31x^-0.081^ (r^2^ = 0.76).

### Trial 2

#### Clinical chemistry and hepatic lipid content

**Fig 4:**
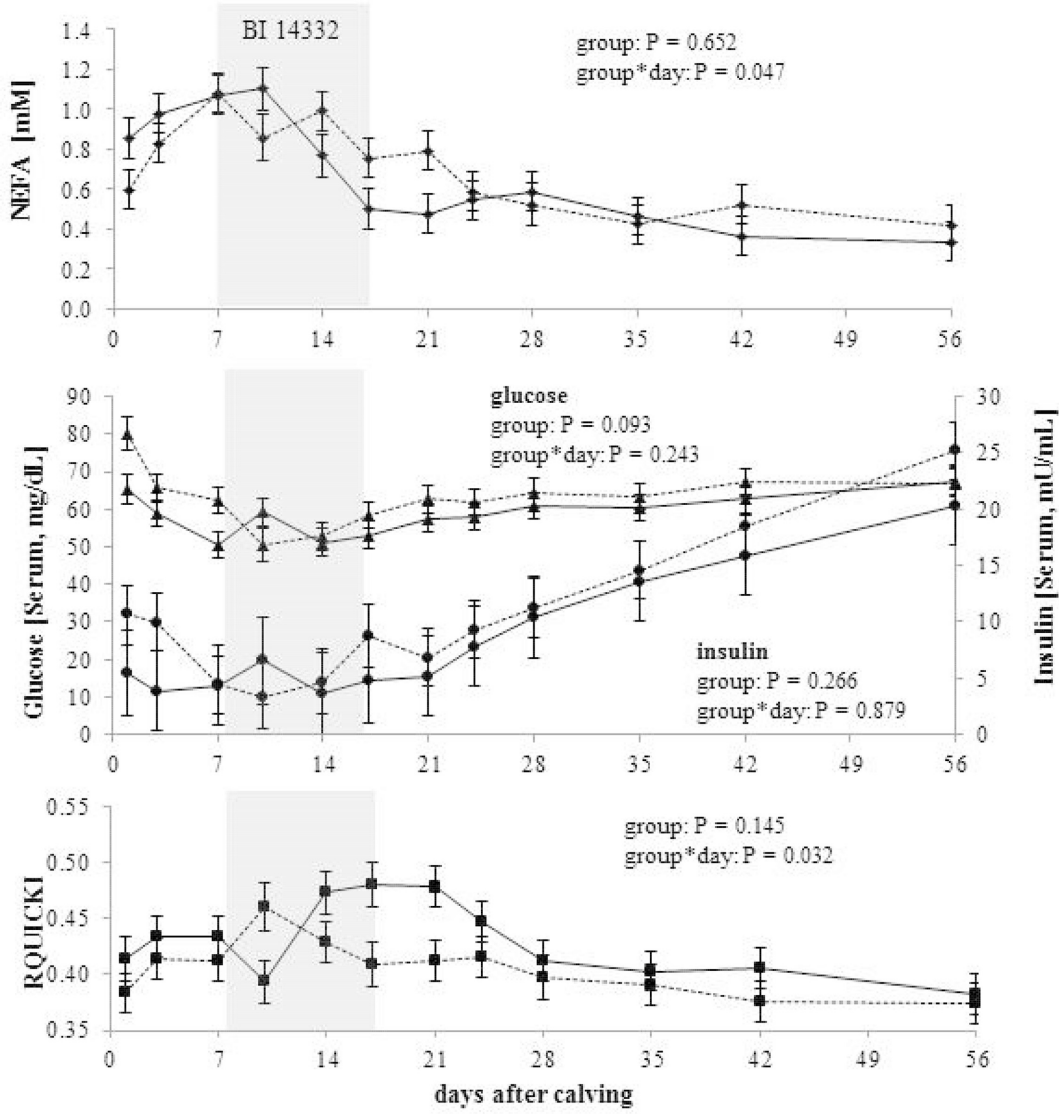
Concentrations of non-esterified fatty acids (NEFA), glucose and insulin in serum, and insulin sensitivity (RQUICKI) in cows with subclinical ketosis. With the first occurrence of serum β-hydroxybutyrate concentrations ≥ 1.2 mM, cows were treated with BI 14332 [(**—**) n = 6] or stayed untreated as control [(···) n = 6]. Within the BI 14332 treatment group subclinical ketosis was diagnosed on day +7 (5 cows) and on day +10 (1 cows), relative to calving. Dosage of BI 14332 was 0.3 mg/kg body weight, applied i.v. once a day over a period of 7 days. The statistical analysis included group (BI 14332 treatment vs. control), experimental day (1^st^ day post partum until 56^th^ day post partum), and the interaction (*P* < 0.05, Tukey test). Experimental day differed significantly for all parameters. [NEFA (diamond), Glucose (triangle), Insulin (circle), RQUICKI (square)].

An overview of the clinical chemical parameters is given in Table 3. A significant group*time interaction was shown for NEFA, TG and GLDH. All three variables changed significantly with time and were additionally influenced by treatment. For TG, this was indicated by a significantly lower concentration in the HC-BI cows during the 1^st^ week after treatment compared to the HC-Con cows. For NEFA, there was a significant decrease in the concentration between the week of treatment to the 1^st^ and the 2^nd^ week post treatment, only within the HC-BI group, while GLDH increased significantly in the HC-Con cows and peaked in the 2^nd^ week post observation. Further time-dependent alterations were detected for glucose, insulin, AST and γ-GT. Glucose and insulin concentrations were significantly greater two weeks post observation than during observation in the HC-Con group. For more detailed evaluation of variables describing glycemic control, Fig 4 shows RQUICKI and the variables necessary to calculate the index (NEFA, glucose, insulin). The figure reveals significant changes relative to calving and in accordance to treatment. On day +10 post partum, NEFA, glucose and insulin peaked within the HC-BI group, while RQUICKI decreased to a nadir at that day. For the HC-Con cows there was the opposite effect as substantiated by the significant interaction between group and day for RQUICKI. Starting on day +14 until day +21 post partum, RQUICKI differed markedly between the experimental groups, with higher values for the HC-BI cows.

**Table 3:** Effects of dipeptidyl peptidase-4 (DPP4) inhibition via BI 14332 to blood serum variables of clinical chemistry and insulin sensitivity of cows with subclinical ketosis (LSmeans ±SE).

	**HC-BI (n = 6)** ^a^				**HC-Con (n = 6)** ^a^				**Probability**		
Parameter	Day of classification^b^	Treatment	1^st^ week after treatment	2^nd^ week after treatment	Day of classification^b^	Observation	1^st^ week after observation	2^nd^ week after observation	group	time	Group x time
BHB [mM]	1.63 ± 0.65	1.77 ± 0.38	1.07 ± 0.28	1.56 ± 0.31	1.51 ± 0.23	1.44 ± 0.31	1.15 ± 0.30	1.12 ± 0.32	0.509	0.166	0.520
NEFA [mM]	0.92 ± 0.28	1.09 ± 0.10	0.53 ± 0.07	0.56 ± 0.08	0.94 ± 0.23	0.94 ± 0.08	0.75 ± 0.08	0.55 ± 0.08	0.820	**< 0.001**	**0.026**
Triglyceride [mg/dL]	11.07 ± 2.40	11.20 ± 1.10	9.03 ± 0.74	9.57 ± 0.85	12.34 ± 3.69	11.37 ± 0.87	14.70 ± 0.81	11.02 ± 0.88	**0.014**	0.123	**0.002**
Glucose [mg/dL]	50.68 ± 9.22	55.09 ± 4.49	57.64 ± 3.72	59.86 ± 3.97	63.05 ± 8.52	51.70 ± 3.93	60.84 ± 3.74	62.58 ± 3.89	0.876	**0.018**	0.404
Insulin [mU/mL]	5.20 ± 2.72	4.58 ± 1.79	5.78 ± 1.29	8.63 ± 1.44	6.70 ± 5.62	5.49 ± 1.46	7.17 ± 1.43	11.21 ± 1.48	0.313	**0.001**	0.802
RQUICKI	0.44 ± 0.04	0.43 ± 0.02	0.47 ± 0.01	0.44 ± 0.02	0.41 ± 0.04	0.43 ± 0.02	0.42 ± 0.02	0.39 ± 0.02	0.129	0.127	0.160
Haptoglobin [mg/mL]	1.56 ± 1.63	0.31 ± 0.30	0.13 ± 0.21	0.12 ± 0.23	1.27 ± 1.22	0.76 ± 0.24	0.53 ± 0.23	0.32 ± 0.24	0.168	0.385	0.817
AST [U/I]	108.03 ± 35.3	117.00 ± 10.8	104.86 ± 8.18	86.43 ± 8.92	96.79 ± 21.0	112.65 ± 9.07	105.11 ± 8.68	92.83 ± 9.19	0.941	**0.005**	0.772
γ-GT [U/I]	18.49 ± 3.41	21.67 ± 9.01	31.50 ± 8.15	40.30 ± 8.37	19.95 ± 4.18	22.94 ± 8.44	28.54 ± 8.30	38.85 ± 8.47	0.928	**0.001**	0.868
**GLDH [U/I]**	**8.13 ± 1.56**	**19.89 ± 7.72**	**29.75 ± 6.56**	**27.08 ± 6.88**	**9.42 ± 2.59**	**13.59 ± 6.90**	**20.92 ± 6.67**	**39.41 ± 6.97**	**0.918**	**0.003**	**0.028**

^a^With first occurrence of serum β-hydroxybutyrate (BHB) concentration ≥ 1.2 mM cows were treated with BI 14332 (HC-BI) or stayed untreated as control group (HC-Con). BI 14332 was applied once a day over a period of 7 days (i.v., 0.3 mg/kg body weight). Subclinical ketosis was diagnosed on day +3 (1 cow), day +7 (8 cows) or on day +10 (3 cows) after calving.

^b^The day of classification (mean ± SD), which was the day with first occurrence of BHB values ≥ 1.2 mM were set as covariate, integrated in the MIXED procedure of SAS [24] with group and time as fixed factors (P ≤0.05; Tukey test). Significant values are shown in bold.

NEFA, non-esterified fatty acids; RQUICKI, revised quick insulin sensitivity index; AST, aspartate aminotransferase; γ-GT, γ-glutamyltransferase; GLDH, glutamate dehydrogenase

The BHB concentrations were not different between the groups and the same was true for Hp. The greatest Hp concentrations were detected at the day of classification in both groups; thereafter the concentration decreased continuously. Albeit not reaching the level of significance, the decrease of the Hp concentration seemed to be faster in HC-BI than in the HC-Con cows.

There was also no significant difference for the total liver lipid content between groups (data not shown), even though HC-Con cows had slightly higher lipid contents than the HC-BI cows at all days evaluated post partum (Δ = 13.8 mg/g). For both groups the total hepatic lipid content differed significantly with time and highest contents were detected on day +7 after calving with 161 mg/g (HC-BI) and 175 mg/g (HC-Con).

#### DMI and milk yield

Statistical evaluation of dry matter intake and milk yield during lactation is shown in Table 4. Data offered a significant time effect for the variables. Milk yield and DMI increased continuously for both subgroups. A group effect, respectively a significant group*time interaction, was not determined for the named parameters.

**Table 4:** Effects of dipeptidyl peptidase IV inhibition via BI 14332 to dry matter intake (DMI) and milk yield of cows with subclinical ketosis (LSmeans ± SE).

	**HC-BI (n = 6)** ^a^				**HC-Con (n = 6)** ^a^				**Probability**		
Parameter	Covariate^b^	2^nd^ week of lactation	3^rd^ and 4^th^ week of lactation	5^th^ until 8^th^ week of lactation	Covariate^b^	2^nd^ week of lactation	3^rd^ and 4^th^ week of lactation	5^th^ until 8^th^ week of lactation	group	time	groupx time
DMI [kg/d]	13.3 ± 2.0	13.2 ± 0.7	16.5 ± 0.6	20.4 ± 0.5	12.6 ± 2.6	14.6 ± 0.8	17.4 ± 0.6	20.5 ± 0.5	0.240	**0.001**	0.400
**Milk yield [kg/d]**	**30.7 ± 5.7**	**31.0 ± 1.7**	**36.5 ± 1.6**	**39.1 ± 1.5**	**30.2 ± 4.5**	**31.2 ± 1.5**	**34.5 ± 1.4**	**37.4 ± 1.4**	**0.592**	**0.001**	**0.150**

^a^With first occurrence of serum β-hydroxybutyrate (BHB) concentration ≥ 1.2 mM cows were treated with BI 14332 (HC-BI) or stayed untreated as control group (HC-Con). BI 14332 was applied once a day over a period of 7 days (intravenous, 0.3 mg/kg body weight). Subclinical ketosis was diagnosed during 1st and 2nd week of lactation (day +3, day +7 or day +10 after calving).

^b^The first week (mean ± SD) was set as covariate, integrated in the MIXED procedure of SAS [24] with group and time as fixed factors (P ≤0.05; Tukey test).

#### Hematology and cell function

Variables of the hematology and the SI of PBMC are shown in Table 5. White blood cells (WBC) were significantly influenced by time and time*group. During treatment, leucocytes and granulocytes counts were significantly higher in the HC-BI than in the HC-Con group. The decrease of the named parameters from treatment to the 1^st^ and the 2^nd^ week after treatment was significant in the HC-BI group. The proliferative capability of PBMC in the ex vivo assay did not change over time. T-cell-phenotyping revealed that the CD4^+^/CD8^+^ ratio of subclinically cows tended to be higher for the HC-Con versus the HC-BI group (*P* = 0.059, Table 6). For the HC-Con cows the increase from ante partum to treatment period was significant; due to a selective increase of the CD4^+^ T-cell population (~ 11%) and a slight decrease of the CD8^+^ T-cell population (~ 10%). In the HC-BI group, both subpopulations decreased (CD4^+^: ~ 16%, CD8^+^: ~ 26%) during treatment. Both experimental groups showed significant time effect with highest ratios during observation (HC-Con: 3.28) and two weeks after treatment (HC-BI: 2.60). The individual CD4^+^ and CD8^+^ cell populations differed significantly over the time, but neither group nor the group*time interaction were significant.

**Table 5:** Effects of dipeptidyl peptidase-4 (DPP4) inhibition via BI 14332 to hematological variables and the proliferative capability of PBMC (LSmeans ± SE).

	**HC-BI (n = 6)** ^a^				**HC-Con (n = 6)** ^a^				**Probability**		
Parameter	Day of Classification^b^	Treatment	1^st^ week after treatment	2^nd^ week after treatment	Day of Classification^b^	Observation	1^st^ week after observation	2^nd^ week after observation	group	time	Group x time
SI	6.69 ± 1.29	6.05 ± 0.79	6.26 ± 0.56	3.69 ± 1.42	7.27 ± 1.62	6.73 ± 0.75	6.49 ± 0.64	6.80 ± 1.37	0.138	0.591	0.453
Leucocytes [10^3^/μL]	9.75 ± 2.79	10.17 ± 0.65	7.59 ± 0.47	7.14 ± 0.51	7.35 ± 3.07	7.33 ± 0.52	7.48 ± 0.50	7.05 ± 0.56	0.105	**0.005**	**0.009**
LY [10^3^/μL]	3.30 ± 1.07	2.79 ± 0.22	2.86 ± 0.19	2.89 ± 0.20	2.93 ± 0.41	2.95 ± 0.20	2.97 ± 0.19	3.09 ± 0.20	0.549	0.642	0.927
GR [10^3^/μL]	5.87 ± 2.01	6.74 ± 0.66	4.01 ± 0.48	3.75 ± 0.52	3.77 ± 2.95	4.00 ± 0.53	3.99 ± 0.51	3.37 ± 0.57	0.106	**0.002**	**0.015**
EO [10^3^/μL]	0.40 ± 0.24	0.33 ± 0.12	0.39 ± 0.09	0.29 ± 0.10	0.45 ± 0.26	0.27 ± 0.10	0.34 ± 0.10	0.40 ± 0.11	0.987	0.730	0.426
Erythrocytes [10^6^/μL]	5.92 ± 0.42	5.81 ± 0.21	5.69 ± 0.19	5.53 ± 0.20	6.07 ± 0.82	5.90 ± 0.20	5.94 ± 0.19	5.87 ± 0.20	0.407	0.238	0.468
HGB [g/dL]	10.82 ± 0.68	10.23 ± 0.42	9.90 ± 0.37	9.69 ± 0.39	10.52 ± 1.18	10.40 ± 0.39	10.38 ± 0.39	10.31 ± 0.40	0.435	0.331	0.551
HCT [%]	36.30 ± 2.61	34.60 ± 1.36	33.48 ± 1.20	32.27 ± 1.24	35.07 ± 4.09	34.70 ± 1.26	34.59 ± 1.24	34.43 ± 1.30	0.510	0.186	0.347
**Platelets [10^3^/μL]**	**514 ± 127**	**117 ± 653**	**1029 ± 515**	**1631 ± 550**	**453 ± 48**	**835 ± 561**	**862 ± 538**	**909 ± 583**	**0.933**	**0.211**	**0.276**

^a^With first occurrence of serum β-hydroxybutyrate ≥ 1.2 mM cows were treated with BI 14332 (HC-BI) or stayed untreated as control (HC-Con). BI 14332 was applied once a day over a period of 7 days (intravenous, 0.3 mg/kg body weight). Subclinical ketosis was diagnosed on day +3 (1 cow), day +7 (8 cows) or on day +10 (3 cows) after calving.

^b^The day of classification (mean ± SD), which was the day with first occurrence of BHB values ≥ 1.2 mM were set as covariate, integrated in the MIXED procedure of SAS [24] with group and time as fixed factors (P ≤0.05; Tukey test). Significant values are shown in bold.

SI, stimulation index (ratio between the fluorescence in the Alamar Blue assay of concanavalin A-stimulated and unstimulated PBMC); LY, lymphocytes; GR, granulocytes; EO, eosinophile granulocytes; HGB, hemoglobin; HCT, hematocrit

**Table 6:** Effects of dipeptidyl peptidase IV (DPP4) inhibition via BI 14332 to relative numbers of CD4^+^ and CD8^+^ T-lymphocytes (LSmean ± SE).

	**HC-BI (n = 6)** ^a^				**HC-Con (n = 6)** ^a^				**Probability**		
Parameter	Ante partum	Treatment	2 weeks after treatment	End of trial	Ante partum	Observation	2 weeks after observation	End of trial	group	time	Group x time
CD4^+^ [%]	28.8 ± 2.3^a^ ^b^	24.0 ± 2.9^a^	33.5 ± 2.0^b^	34.0 ± 2.37^b^	28.8 ± 2.1^a^	32.4 ± 2.7^a^ ^b^	33.2 ± 2.4^a^ ^b^	36.5 ± 2.2^b^	0.314	**0.001**	0.155
CD8^+^ [%]	15.6 ± 1.2	11.6 ± 1.6	13.7 ± 1.1	14.4 ± 1.30	12.9 ± 1.2	11.5 ± 1.5	11.7 ± 1.3	13.6 ± 1.2	0.347	**0.045**	0.535
**CD4^+^/CD8^+^**	**1.85 ± 0.25**	**2.06 ± 0.33**	**2.60 ± 0.22**	**2.46 ± 0.27**	**2.29 ± 0.24^a^**	**3.28 ± 0.30^b^**	**2.90 ± 0.26^a^**	**2.83 ± 0.25^a^**	**0.059**	**0.001**	**0.245**

^a^With first occurrence of serum β-hydroxybutyrate ≥ 1.2 mM cows were treated with BI 14332 (HC-BI) or stayed untreated as control group (HC-Con). BI 14332 was applied once a day over a period of 7 days (0.3 mg/kg body weight). Subclinical ketosis was diagnosed on day +3 (1 cow), day +7 (8 cows) or on day +10 (3 cows) after calving. Significant values (P ≤ 0.05) and trends (P ≤ 0.1) are shown in bold. LSmeans with different superscripts (a-b) within the same group are significantly different.

## Discussion

Most of the findings about inhibition of DPP4 are related to type II diabetes in human patients, opening up new perspectives in therapy. The ketotic metabolic status of high yielding dairy cows during early lactation is characterized by some similarities with the metabolic situation of type II diabetes and fatty liver in humans [29].

The first aims of the current investigation were (1) to show that BI 14332 is an effective DPP4 inhibitor and (2) to find the optimal dosage of BI 14332. Therefore, all doses resulted in an inhibition of DPP4 activity in plasma and liver without any indication for adverse side-effects. According to the PK/PD results, we considered the dosage of 0.3 mg/kg BW as most suitable. Albeit the dosage of 3 mg/kg BW yielded the highest AUC of BI 14332, the plasma AUC of DPP4 activity was quite similar in the highest and the lowest dosage group. The terminal t_1/2_ is the time required to halve the plasma concentration after reaching a steady state equilibrium and has to be known to provide an appropriate length for the dosing interval [30], which was sought to be 24 h. Results confirm dosing decision of 0.3 mg/kg BW, as t_1/2_ and Cl_24h_, i.e. the ability to eliminate a drug [31], offered the longest retention period in blood.

The aim of trial 2 was to test whether inhibiting DPP4 may affect blood variables associated with lipid metabolism and glycemic control under catabolic condition. The results showed that the impact of DPP4 inhibition was less in cows of subclinical ketosis, respectively during early lactation. Significant changes and positive impacts were limited to the time after treatment (Table 3; NEFA, TG, GLDH). Lower TG concentrations were also observed by Ben-Shlomo et al. [3]. The researches show significant reduced levels in DPP4-deficient rats and explain a GLP-1 mechanism in liver which induces a signal for a low energy state. Therefore, protein kinase activities involved in the pathway effect a reduced expression of lipogenesis-related genes. Effects of DMI and milk yield showed that there was no difference between the subgroups (Table 4), which could have explained a lower lipolysis for the HC-BI cows. Therefore, the decrease in NEFA was not caused by a higher DMI or a lower milk yield for those cows compared to the HC-Con cows.

RQUICKI, as surrogate marker to assess insulin sensitivity, includes NEFA in the equation and the lower NEFA concentrations in HC-BI vs. HC-Con rather than the ones of glucose and insulin affected RQUICKI. However, differences between the groups were limited to diverging reaction over time, i.e. the interaction of time and treatment. At day +14 post partum RQUICKI was greater in the BI 14332 treated animals. At this stage, approximately during the middle of the treatment, a steady state plateau is reached and therapeutic efficacy is assumingly complete [32]. In a further study, RQUICKI remained unaffected during subclinical ketosis [19]. We conclude, that RQUICKI may be not sensitive enough to investigate alterations of insulin sensitivity in case of subclinical ketosis, but it is more likely that insulin sensitivity is not, or even very less, influenced by subclinical ketosis and a diminished sensitivity is limited to physiological changes around calving. Nevertheless RQUICKI was sensitive enough to show a transient improvement of insulin sensitivity by BI 14332. Taking into account that DPP4 inhibition was significant (Fig 2, Table 2) and with respect to investigations made in humans and rodents, one could assume that the retention period of active GLP-1 in the periphery was prolonged. If that is also true in cows, it may explain the positive impacts on lipaemic control. However, the support was not strong enough to affect the entire metabolism.

The fact that effects of DPP4 inhibition were marginal may be linked to the short duration of the treatment period [2]; which was possibly reflected by GLDH. Changes in GLDH became evident only late [19,33] and indicated less hepatic lesions in the second week after BI 14332 treatment. This was not evidenced by a reduced level of hepatic fat in the HC-BI cows. A prolonged treatment period might affect hepatic infiltrations of lipids in a stronger way.

Another aim of trial 2 was to investigate if the inhibition of DPP4 via BI 14332 has immune-modulating effects. The prescribing information of sitagliptin, the first DPP4 inhibitor for clinical use, reports a slight increase in WBC, primarily due to a small increase of neutrophil granulocytes counts [34]. The HC-BI cows had already higher WBC values before the onset of the treatment, with Δ = 2.40 · 10^3^/μl at the day of classification compared to cows of control group. Therefore, it was questionable, if significant changes of WBC and GR counts were indeed related to BI 14332 or just coincided with time-related alterations around calving. Our investigations regarding the proliferative capability of PBMC showed similar results and immune-modulatory alterations were not evident. Studies employing DPP4 inhibitors showed that T-cell proliferation and cytokine production is inhibited by impaired DNA synthesis [35]. In contrast, Anz et al. [36] showed results, similar to the present. None of the tested DPP4 inhibitors impaired key parameters of the innate and adaptive immune response, which were included in the present study to assess drug safety.

For the current investigation, immune-modulatory effects were limited to differences in CD4^+^/CD8^+^ ratio, which tended to be higher for the HC-Con cows (vs. HC-BI cows), with a significant increase after calving (~ 30%) up to 3.28. A ratio up to 2.5 suggests a physiological situation, while an increased ratio may indicate an immune dysregulation [37,38]. Furthermore it is known that DPP4 is expressed predominantly on T-lymphocytes and most of the T-cells expressing DPP4 belong to the CD4^+^ population (~ 56%) [39]. The inhibition of DPP4 activity may lower the expression of CD4^+^ and CD8^+^ T-cells. This was more pronounced for CD8^+^ cells and led to a better CD4^+^/CD8^+^ ratio. It suggests an impaired immune defense after calving, when the need for an appropriate defense is highest. In view of the concentrations of the acute-phase protein Hp that is commonly used as marker of inflammation, a beneficial effect of BI 14332 on the immune defense was not supported. Haptoglobin is assumed to be elevated by tissue lesions occurring during birth and by the general proinflammatory situation for the time around calving [40]. It positively correlates with BHB, NEFA and TG [10,24,41]. The minor impact of the DPP4 inhibition regarding these variables may explain for the insignificant differences in Hp concentrations between groups. Nevertheless, in numerical terms alone, within the HC-BI group Hp dropped markedly by about 80% (vs. ~ 40% for HC-Con cows; Table 3) during the treatment. Thus a prolonged treatment period together with a concomitant improvement of hepatic lipid metabolism may also reduce Hp.

## Conclusions

The DPP4 activity was determined in plasma and liver samples of dairy cows. The DPP4 inhibitor BI 14332 reduced the enzymatic activity in vivo and showed a fast onset and a long lasting inhibition of DPP4. However, the DPP4 inhibition did not improve the metabolic disarrangements related to subclinical ketosis. Albeit an improved lipaemic control was observed, as NEFA and TG were decreased after treatment. Unfortunately, the support was not strong enough to affect main markers of ketosis (BHB, hepatic lipid content).

## References

[1] Deacon CF. Circulation and degradation of GIP and GLP-1. Horm Metab Res 2004; 36: 761 765. 15655705 10.1055/s-2004-826160

[2] Drucker DJ, Nauck MA. The incretin system: glucagon-like peptide-1 receptor agonists and dipeptidyl peptidase-4 inhibitors in type 2 diabetes. Lancet 2006; 368: 1696–1705. 17098089 10.1016/S0140-6736(06)69705-5

[3] Ben-Shlomo S, Zvibel I, Shnell M, Shlomai A, Chepurko E, Halpern Z et al (2011) Glucagon-like peptide-1 reduces hepatic lipogenesis via activation of AMP-activated protein kinase. J Hepatol 54: 1214–1223. 10.1016/j.jhep.2010.09.032 21145820

[4] Flatt PR, Bailey CJ, Green BD. Dipeptidyl peptidase IV (DPP IV) and related molecules in type 2 diabetes. Front Biosci 2008; 13: 3648–3660. 18508462 10.2741/2956

[5] Bell AW, Bauman DE. Adaptations of glucose metabolism during pregnancy and lactation. J Mammary Gland Biol Neoplasia 1997; 2: 265–278. 10882310 10.1023/a:1026336505343

[6] Hove K. Insulin secretion in lactating cows: responses to glucose infused intravenously in normal, ketonemic, and starved animals. J Dairy Sci 1978; 61: 1407–1413. 361768 10.3168/jds.S0022-0302(78)83742-4

[7] Ohtsuka H, Koiwa M, Hatsugaya A, Kudo K, Hoshi F, Itoh N et al Relationship between serum TNF activity and insulin resistance in dairy cows affected with naturally occurring fatty liver. J Vet Med Sci 2001; 63: 1021–1025. 11642272 10.1292/jvms.63.1021

[8] McArt JA, Nydam DV, Oetzel GR, Overton TR, Ospina PA. Elevated non-esterified fatty acids and beta-hydroxybutyrate and their association with transition dairy cow performance. Vet J 2013; 198: 560–570. 10.1016/j.tvjl.2013.08.011 24054909

[9] Bobe G, Young JW, Beitz DC. Invited review: pathology, etiology, prevention, and treatment of fatty liver in dairy cows. J Dairy Sci 2004; 87: 3105–3124. 15377589 10.3168/jds.S0022-0302(04)73446-3

[10] Saremi B, Al-Dawood A, Winand S, Müller U, Pappritz J, von Soosten D et al Bovine haptoglobin as an adipokine: serum concentrations and tissue expression in dairy cows receiving a conjugated linoleic acids supplement throughout lactation. Vet Immunol Immunopathol 2012; 146: 201–211. 10.1016/j.vetimm.2012.03.011 22498004

[11] Saremi B, Winand S, Friedrichs P, Kinoshita A, Rehage J, Dänicke S et al Longitudinal profiling of the tissue-specific expression of genes related with insulin sensitivity in dairy cows during lactation focusing on different fat depots. PLoS One 2014; 9: e86211 10.1371/journal.pone.0086211 24465964 PMC3897665

[12] Taylor-Edwards CC, Burrin DG, Matthews JC, McLeod KR, Holst JJ, Harmon DL. Expression of mRNA for proglucagon and glucagon-like peptide-2 (GLP-2) receptor in the ruminant gastrointestinal tract and the influence of energy intake. Domest Anim Endocrinol 2010; 39: 181–193. 10.1016/j.domaniend.2010.05.002 20688461

[13] Larsen M, Relling AE, Reynolds CK, Kristensen NB. Effect of abomasal glucose infusion on plasma concentrations of gut peptides in periparturient dairy cows. J Dairy Sci 2010; 93: 5729–5736. 10.3168/jds.2010-3258 21094744

[14] Relling AE, Reynolds CK. Abomasal infusion of casein, starch and soybean oil differentially affect plasma concentrations of gut peptides and feed intake in lactating dairy cows. Domest Anim Endocrinol 2008; 35: 35–45. 10.1016/j.domaniend.2008.01.005 18308502

[15] Bradford BJ, Harvatine KJ, Allen MS. Dietary unsaturated fatty acids increase plasma glucagon-like peptide-1 and cholecystokinin and may decrease premeal ghrelin in lactating dairy cows. J Dairy Sci 2008; 91: 1443–1450. 10.3168/jds.2007-0670 18349237

[16] Pezeshki A, Muench GP, Chelikani PK. Short communication: expression of peptide YY, proglucagon, neuropeptide Y receptor Y2, and glucagon-like peptide-1 receptor in bovine peripheral tissues. J Dairy Sci 2012; 95: 5089–5094. 10.3168/jds.2011-5311 22916913

[17] Connor EE, Baldwin RL, Capuco AV, Evock-Clover CM, Ellis SE, Sciabica KS. Characterization of glucagon-like peptide 2 pathway member expression in bovine gastrointestinal tract. J Dairy Sci 2010; 93: 5167–5178. 10.3168/jds.2010-3205 20965332

[18] Starke A, Haudum A, Busche R, Beyerbach M, Dänicke S, Rahge J. Technical note: Analysis of total lipid and triacylglycerol content in small liver biopsy samples in cattle. J Anim Sci 2010; 88: 2741–2750. 10.2527/jas.2009-2599 20348378

[19] Schulz K, Frahm J, Meyer U, Kersten S, Reiche D, Rehage J et al Effects of prepartal body condition score and peripartal energy supply of dairy cows on postpartal lipolysis, energy balance and ketogenesis: an animal model to investigate subclinical ketosis. J Dairy Res 2014; 81: 257–266. 10.1017/S0022029914000107 24594287

[20] Duffield T. Subclinical ketosis in lactating dairy cattle. Vet Clin North Am Food Anim Pract 2000; 16: 231–253. 11022338 10.1016/s0749-0720(15)30103-1

[21] Edmonson AJ, Lean IJ, Weaver LD, Farver T, Webster G. A Body Condition Scoring Chart for Holstein Dairy-Cows. Journal of dairy science 1989; 72: 68–78.

[22] Huttner S, Graefe-Mody EU, Withopf B, Ring A, Dugi KA. Safety, tolerability, pharmacokinetics, and pharmacodynamics of single oral doses of BI 1356, an inhibitor of dipeptidyl peptidase 4, in healthy male volunteers. J Clin Pharmacol 2008; 48: 1171–1178. 10.1177/0091270008323753 18812608

[23] Hiss S, Mielenz M, Bruckmaier RM, Sauerwein H. Haptoglobin concentrations in blood and milk after endotoxin challenge and quantification of mammary Hp mRNA expression. J Dairy Sci 2004; 87: 3778–3784. 15483161 10.3168/jds.S0022-0302(04)73516-X

[24] Schulz K, Frahm J, Kersten S, Meyer U, Reiche D, Sauerwein H et al Effects of elevated parameters of subclinical ketosis on the immune system of dairy cows: in vivo and in vitro results. Arch Anim Nutr 2015; 69: 113–127. 10.1080/1745039X.2015.1013666 25708603

[25] Stelter K, Frahm J, Paulsen J, Berk A, Kleinwachter M, Selmar D et al Effects of oregano on performance and immunmodulating factors in weaned piglets. Arch Anim Nutr 2013; 67: 461–476. 10.1080/1745039X.2013.858897 24228909

[26] StatSoft I. STATISTICA for Windows Operating System (Version 10.0). 2011 Tulsa (OH): StatSoft.

[27] Holtenius P, Holtenius K. A model to estimate insulin sensitivity in dairy cows. Acta Vet Scand 2007; 49: 29 17931417 10.1186/1751-0147-49-29 PMC2092429

[28] SAS Institute. The SAS/STAT 9.1 User's Guide. 2004; Vol. 1–7 SAS Institute Cary, NC.

[29] Tilg H, Moschen AR. Insulin resistance, inflammation, and non-alcoholic fatty liver disease. Trends Endocrinol Metab 2008; 19: 371–379. 10.1016/j.tem.2008.08.005 18929493

[30] Toutain PL, Bousquet-Melou A. Plasma terminal half-life. J Vet Pharmacol Ther 2004; 27: 427–439. 15601438 10.1111/j.1365-2885.2004.00600.x

[31] Toutain PL, Bousquet-Melou A. Plasma clearance. J Vet Pharmacol Ther 2004; 27: 415–425. 15601437 10.1111/j.1365-2885.2004.00605.x

[32] Frey H, Löscher W. Lehrbuch der Pharmakologie und Toxikologie für die Veterinärmedizin [Pharmacology and Toxicology in Veterinary Medicine]. Stuttgart: Enke; 2009.

[33] Dirksen G, Gründer H, Stöber M. Die klinische Untersuchung des Rindes [The clinical investigation of bovine animals] Stuttgart: Enke; 2012.

[34] White JR. Dipeptidyl peptidase-IV inhibitors: pharmacological profile and clinical use. Clin Diab 2008; 26: 53–57.

[35] Gorrell MD, Gysbers V, McCaughan GW. CD26: a multifunctional integral membrane and secreted protein of activated lymphocytes. Scand J Immunol 2001; 54: 249–264. 11555388 10.1046/j.1365-3083.2001.00984.x

[36] Anz D, Kruger S, Haubner S, Rapp M, Bourquin C, Endres S. The dipeptidylpeptidase-IV inhibitors sitagliptin, vildagliptin and saxagliptin do not impair innate and adaptive immune responses. Diabetes Obes Metab 2014; 16: 569–572. 10.1111/dom.12246 24320733

[37] Mehrzad J, Zhao X. T lymphocyte proliferative capacity and CD4+/CD8+ ratio in primiparous and pluriparous lactating cows. J Dairy Res 2008; 75: 457–465. 10.1017/S0022029908003439 18701001

[38] Asai K, Kai K, Rikiishi H, Sugawara S, Maruyama Y, Yamaguchi T et al Variation in CD4+ T and CD8+ T lymphocyte subpopulations in bovine mammary gland secretions during lactating and non-lactating periods. Vet Immunol Immunopathol 1998; 65: 51–61. 9802576 10.1016/s0165-2427(98)00176-7

[39] Munoz E, Blazquez MV, Madueno JA, Rubio G, Pena J. CD26 induces T-cell proliferation by tyrosine protein phosphorylation. Immunology 1992; 77: 43–50. 1356916 PMC1421601

[40] Hachenberg S, Weinkauf C, Hiss S, Sauerwein H. Evaluation of classification modes potentially suitable to identify metabolic stress in healthy dairy cows during the peripartal period. J Anim Sci 2007; 85: 1923–1932. 17468419 10.2527/jas.2006-480

[41] Fathi E, Hamali H, Kaleibar MT. Application of acute phase proteins as indicators of ketosis and their relation to energy metabolites in post-calving dairy cows. International J Rec Sci Res 2013; 4: 842–845.

